# Life-Cycle Assessment of Nd–Fe–B Rare Earth Magnet Production

**DOI:** 10.1021/acsomega.6c01994

**Published:** 2026-06-16

**Authors:** Thamires Martinho Prados, Leda Maria Saragiotto Colpini, Giancarlo Alfonso Lovón-Canchumani

**Affiliations:** † Postgraduation Program in Environmental Engineering and Technology, Federal University of Parana (UFPR), Setor Palotina, Parana 85953128, Brazil; ‡ Federal University of Parana (UFPR), Campus Avançado de Jandaia do Sul, Jandaia do Sul, Parana 86900088, Brazil

## Abstract

Rare-earth elements are strategic materials essential for renewable energy, electronics, and electric mobility. The growing demand emphasizes the need for a sustainable and efficient supply, considering environmental and economic aspects. Among their main applications are neodymium–iron–boron (Nd–Fe–B) permanent magnets, whose production chain presents significant environmental impacts—from mining to final manufacturing. This study assesses the life cycle of Nd–Fe–B magnets in the Brazilian context, following ISO 14040 and ISO 14044 standards. System modeling was performed in Sankey software, and environmental impacts were calculated using SimaPro 9.1 with data from the EcoInvent database and literature sources. Results indicate that mining is highly impactful, particularly during the roasting stage, due to high energy consumption and emissions of particulate matter and greenhouse gases. In oxide production, leaching and preseparation steps stand out for their use of hydrochloric acid, contributing to marine eutrophication and ionizing radiation. Magnet manufacturing, especially machining, shows major impacts on global warming, ozone depletion, and water use. The study highlights the need for mitigation strategies, such as stricter regulations, circular economy initiatives, and cleaner technologies. Incorporating renewable energy and improving ore processing efficiency can significantly reduce the carbon footprint of Nd–Fe–B magnets.

## Graphical abstract

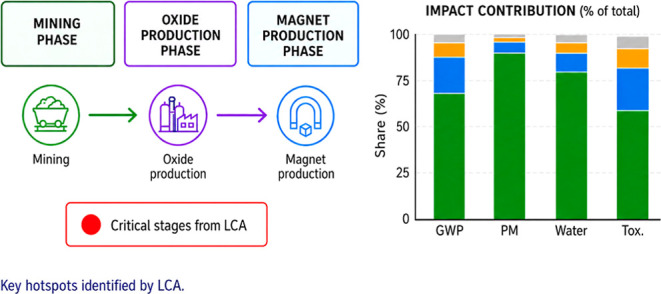

## 1 Introduction

Rare-earth elements (REEs) are mainly composed of lanthanides and have similar physical and chemical properties, which make them essential for various technological and industrial applications. These elements play a key role in the transition to clean energy and are widely used in the manufacture of wind turbines, solar panels, and electric vehicle engines.^1–4^ REEs are classified into two categories: light rare earths (cerium, lanthanum, praseodymium, neodymium, promethium, europium, gadolinium, and samarium) and heavy rare earths (dysprosium, yttrium, terbium, holmium, erbium, thulium, ytterbium, and lutetium).^5^

The growing demand for REEs is directly related to their use in advanced technology products, mainly in the production of neodymium–iron–boron (Nd–Fe–B) permanent magnets.^6,7^ These magnets are significantly stronger than traditional magnets, making it possible to reduce the size of devices without compromising the performance. Currently, the demand for Nd–Fe–B represents around 20% of the total REE consumed globally.^8^ However, worldwide dependence on these elements is a critical factor, since the European Union classifies them as strategic raw materials due to their high economic importance and the risk of supply.^9,10^ Although the global production of REEs is concentrated in China, the lack of production capacity in other countries results in a strong dependence on imports, limiting the progress of various renewable energy technologies.^7^ In view of this restriction, it is essential to explore new sources of REEs and develop innovative methods for their extraction from unconventional materials.^11^

The global dependence on China for the supply of REEs represents a strategic and economic challenge for several countries, especially those seeking to expand their clean technology industries. Despite holding less than 40% of global reserves, China meets more than 60% of international demand for these elements, consolidating its dominance in the production chain.^12^

The growing scarcity of rare earths on the international market has encouraged countries such as Uganda, Australia, Brazil, and Chile to intensify their prospecting and exploration activities for these ores, also driving scientific and technological advances aimed at making more efficient use of these resources.^13^

In addition, diversification of the supply chain is essential to reducing dependence on China, either by exploring new deposits or by developing recycling technologies. The growth of the global rare-earth market, driven by the energy transition and digital transformation, reinforces the need for more responsible and sustainable mining practices, with the implementation of technologies that increase process efficiency and minimize environmental impacts.^14^

The production chain for rare-earth magnets (REMs) involves multiple phases, from ore extraction to final manufacture, and is characterized by significant environmental impacts. Although REEs are available in greater quantities than other industrial metals, their uneven geological distribution and low concentration in economically viable deposits make their utilization an environmental and technological challenge.^11^ Furthermore, the increase in demand for Nd and dysprosium (Dy), which are essential for the production of Nd–Fe–B permanent magnets, reinforces the need for sustainable strategies for their extraction and reuse.^15,16^ It is estimated that by 2050 the demand for these elements will increase by 9 to 35 times in relation to the volumes currently extracted, making it essential to adopt a circular economy model to ensure the sustainability of the supply of these strategic elements for the energy transition.^17–19^

REE extraction presents considerable environmental and social challenges. Conventional mining techniques, such as open-pit and underground mining, demand large amounts of energy, water, and chemicals, contributing to soil degradation, water pollution, and significant atmospheric emissions.^20–22^ The environmental impacts associated with mining include climate change, eutrophication of water bodies, freshwater ecotoxicity, depletion of fossil resources, ionizing radiation, soil acidification, formation of photochemical oxidants, ozone layer degradation, human toxicity, and terrestrial ecotoxicity.^23,24^ During the ore processing, extraction, and separation phase, radionuclides such as uranium and thorium are released, increasing the environmental risk and the challenges of waste management.^21,22,25,26^

Mining and refining operations require high energy consumption and generate greenhouse gas (GHG) emissions.^27–29^ In some operations, the uranium and thorium present in these tailings can be used as byproducts for nuclear power generation, reducing the need for primary mining of these elements and contributing to a low-carbon energy model.^30–32^

Given this scenario, life-cycle assessment (LCA) has emerged as an essential tool for quantifying the environmental impacts of ETRs and proposing mitigation strategies. Standardized by ISO 14040 and ISO 14044, LCA allows the identification of the main critical points in the production cycle, from the extraction of raw materials to final disposal, providing subsidies for the adoption of more sustainable practices.^33,34^ The recycling of rare-earth permanent magnets has been considered a viable alternative for minimizing environmental impacts and reducing dependence on primary extraction.^35^ In addition, the circular economy has gained prominence as a production model that seeks to reduce material waste and encourage the reuse and recycling of these elements.^19^

The LCA of rare-earth mining indicates that energy consumption varies between 0.2 and 1 GJ/t of rare-earth oxides (REO), while water use can reach between 0.3 and 1.8 ML/t REO.^36^ In addition, the environmental impact in terms of GHG emissions is significant, with the process resulting in 1.4 kg of CO_2_-eq/kg of REO produced. The main source of emissions is the use of hydrochloric acid, responsible for around 38% of total emissions, followed by steam consumption (32%) and electricity (12%). Studies indicate that 51% of GHG are due to the use of energy in various forms, such as diesel, steam, fuel oil, and electricity. These figures demonstrate the need to reduce dependence on high-emitting inputs in order to make reproduction more sustainable.^36^

The use of alternative techniques, such as in situ leaching, has been studied as an option for reducing environmental damage.^21,22,37–39^ Studies indicate that environmental impacts vary according to the reagent used, with ammonium sulfate being one of the main contributors to the impacts of the process. Although in situ leaching has less influence on acidification, it contributes significantly to eutrophication. However, this technique, applied to the extraction of clays rich in heavy rare-earth elements (HREEs), reduces environmental impacts and accounts for approximately 35% of the production of these elements in China.^39^

Converting the extracted ore to REO involves physical and chemical processes with a high environmental impact. Minerals such as bastnasite, monazite, and xenotime are the main sources of these oxides, and their processing includes phases of milling, physical beneficiation, chemical separation, and hydrometallurgy.^36,38,40,41^ The hydrometallurgy part of oxides requires large volumes of strong acids, resulting in potentially toxic liquid and solid waste.^38,40^ Depending on the method used, the environmental impacts vary considerably. The production of REO from bastnasite/monazite represents around 35% of the total environmental impacts of the production chain, while extraction based on ion adsorption clays can reach up to 55% of these impacts.^42^

Studies indicate that the production of one ton of REO can result in the emission of 60,000 m^3^ of exhaust gases, which is nothing more than the burning of fuel, as well as 200 m^3^ of acidic wastewater and 1.4 tons of radioactive waste.^43^ Among the most significant environmental impacts associated with the production of REEs is the global warming potential (GWP). The GWP of neodymium (Nd) and dysprosium (Dy) is significantly high, reaching values of 17.6 and 59.6 kg CO_2_-eq/kg, respectively, compared to just 1.5 kg CO_2_-eq/kg for iron.^44^

Nd–Fe–B magnets are essential for various technologies, made up of approximately 29–32% Nd, 1–2% B, and 64–68.5% Fe, standing out for their superior magnetic properties and high efficiency in applications such as electric motors and wind turbines.^45^ However, their production involves significant environmental challenges, since the extraction and magnetization phases consume large amounts of energy and result in the emission of atmospheric pollutants.^46–48^ The durability of magnets varies depending on the application and can be as little as 2 to 3 years in consumer electronics, while in wind turbines it can reach 20 to 30 years.^49–51^

As demand grows, the generation of waste in the production and disposal of magnets has become a critical problem. During manufacture, around 20–30% of the metal alloy is converted into scrap, which reinforces the need for effective recycling processes.^52,53^ Despite this, global recovery rates for rare earths from end-of-life products are less than 1%.^35,51^ The development of new recycling processes is key to minimizing environmental impacts and reducing dependence on primary mining.^54,55^

The production chain for rare-earth magnets (REMs) presents significant environmental challenges, from mining to final manufacture. The high demand for Nd and Dy drives the need for a sustainable production model that minimizes environmental impacts and guarantees the supply of these essential elements for the global energy transition. LCA is an essential tool for identifying and mitigating impacts throughout the life cycle of REEs, providing support for the implementation of more sustainable strategies, such as recycling and the adoption of the circular economy.

The aim of this study is to evaluate the life cycle of Nd–Fe–B magnet production in Brazil, taking into account the specificities of the country’s energy and transportation sectors. Based on this analysis, mitigating measures were proposed to reduce the environmental impacts associated with its production, contributing to a more sustainable development model in the rare-earth sector.

## 2 Methodology

The methodology of this research is structured into three phases, as illustrated in Figure 1. The first phase, of a conceptual nature, involves a bibliographical survey, drawing up the research protocol (form), and pretesting it, resulting in the definition of the research model. The second phase, of practical application, involves technical visits and data collection at different phases of the production process. Finally, the third phase, of direct application, involves modeling the system and carrying out the LCA phases.^33,34^

**Figure 1:**
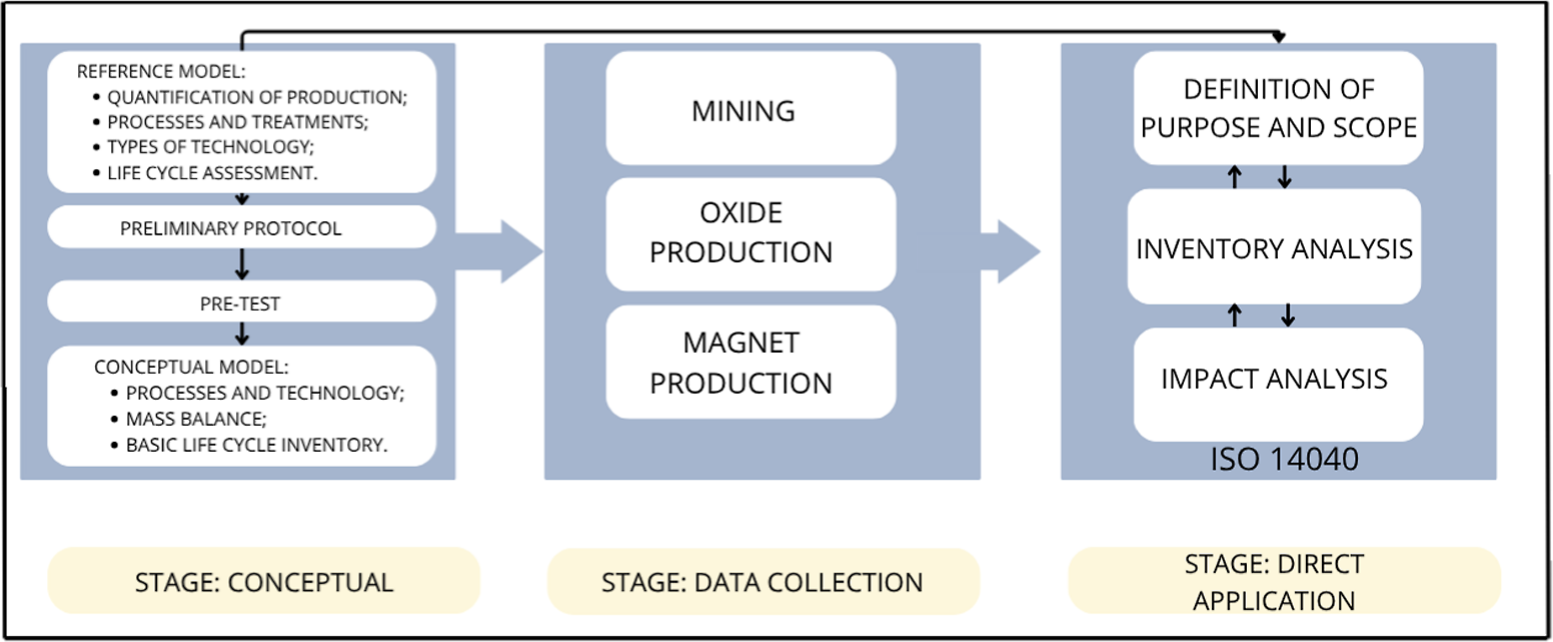
Structure of the study methodology.

Among the methodologies used, LCA stands out for its systemically comprehensive approach. It is a methodology that is widely used to assess the environmental performance of systems, processes, or products, considering all the phases of the life cycle and the respective consumption of resources and associated emissions.^56^ Its systemic approach makes it possible to avoid transferring impacts between different life-cycle phases, geographical regions, or environmental impact categories, providing a more comprehensive and accurate analysis of environmental effects.^57^

The third phase of this research was structured on the basis of ref 58 and the ISO 14040 and ISO 14044 guidelines.^33,34^ To carry out the LCA, four substeps were considered: defining the objective and scope, analyzing the inventory, assessing the impacts, and interpreting the results. The first subphase establishes the purpose of the study and the delimitation of the production chain, defining the limits of the system and its application in the context analyzed. Next, the life-cycle inventory (LCI) analysis quantifies the inputs and outputs of materials and energy throughout the different phases of the production process from mining to REM production. This modeling was carried out with SimaPro software, using data from the literature and the EcoInvent database, adapted to the Brazilian reality. The structure of the material flows was graphically represented in the E! Sankey Software.

### 2.1 Defining the Scope and Objective

The aim of this study is to evaluate the life cycle of REM production, composed of Nd, Fe, and B, considering the Brazilian reality. The main focus is on analyzing the LCI and quantifying the environmental impacts at each phase of the production process. To this end, a production system flow was structured, following the authors,^59^ highlighting the identification of resource inputs and outputs, as well as the associated environmental impacts, from mining to the manufacture of rare-earth magnets, as illustrated in Figure 2.

**Figure 2:**
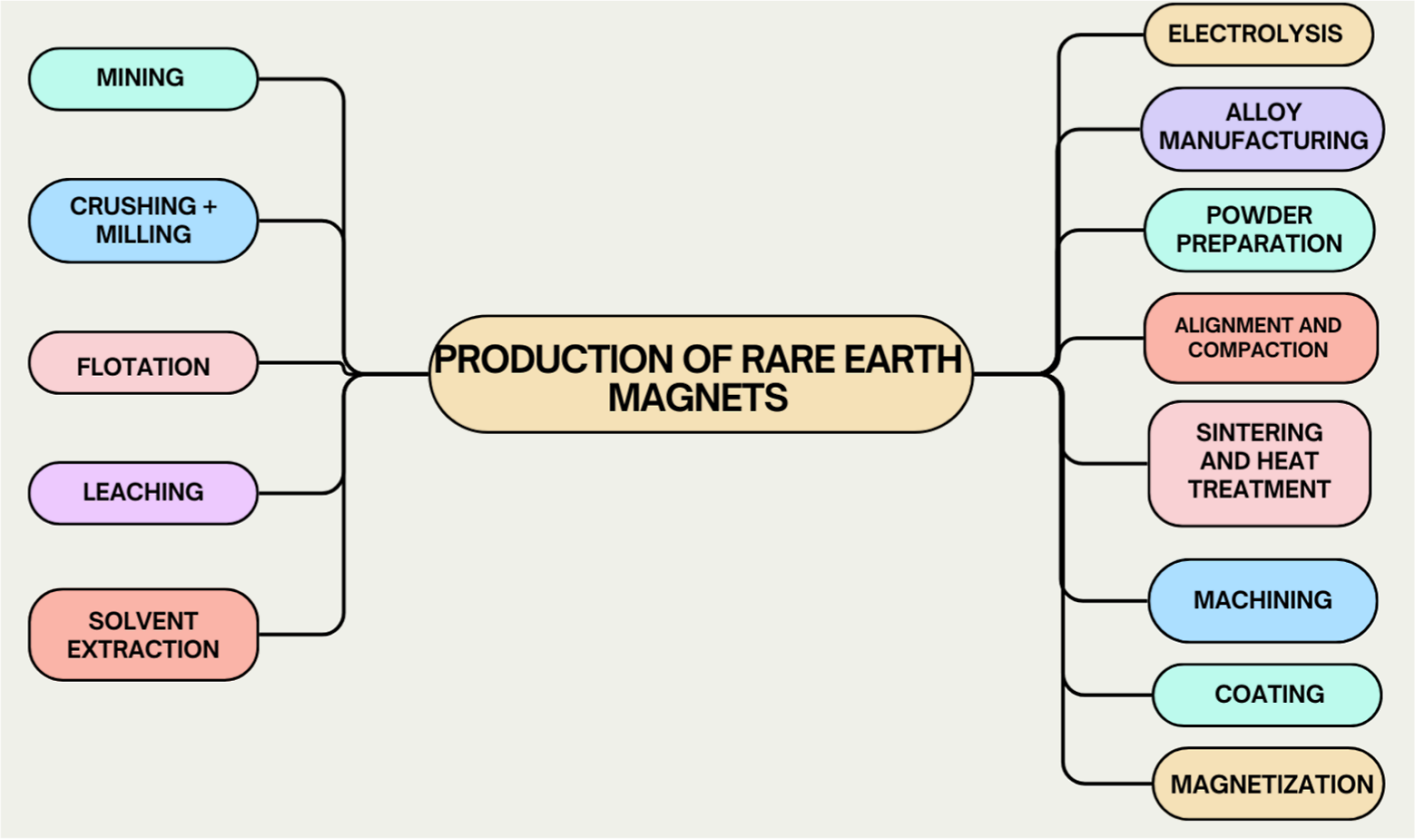
System flow.

The analysis considers aspects such as climate change, toxicity, and energy consumption to assess the environmental impacts of magnet manufacturing in Brazil. Defining system boundaries is essential in LCA, as it determines the processes and flows of materials and energy that have been included in the analysis. In the Brazilian context, these boundaries cover everything from the extraction of raw materials to the manufacture of magnets, including the mining phase, the oxide production phase, and the magnet production phase and, optionally, the distribution or disposal of final products. The inventories that preceded them were adjusted to reflect the national reality, taking into account energy sources and fuels. This delimitation made it possible to quantify the flows at each phase, enabling a detailed assessment of the environmental impacts and opportunities for improvement throughout the life cycle.

#### 2.1.1 Functional Unit

The functional unit adopted for the evaluation corresponds to the production of 1 kg of rare-earth magnets. In order to better represent the Brazilian reality, the analysis was adjusted based on a production plant design of 100 tons, considering the inputs and outputs of the system in a laboratory factory located in the Southeast region of Brazil.

#### 2.1.2 System Modeling

The system was modeled on the basis of a blueprint, allowing data to be entered into the E! Sankey Software. This process made it possible to build process flow diagrams that represent all the production phases, from mining to magnet production, reflecting the particularities of national production. Based on this, the modeling uses arrows with dimensions proportional to the amount of flow, making it easier to see the transfer of materials, energy consumption, and costs between processes.

Figure 3 represents the modeling of the system covering all of the phases of the production process, including mining and each phase within this phase, followed by the production of rare-earth oxides and their phase and, consequently, the production of rare-earth magnets and all of their comprehensive phases. The modeling of the mining system presents a detailed flow of the main phases of the process, from the input of inputs, such as energy, explosive materials, and transport, to the final leaching. The resources used include water, electricity, chemicals, and metals, while the outputs reflect the generation of solid waste, dust, gases, and tailings. The connections between the phases—mining, crushing and milling, flotation, roasting, and leaching—show the continuous flow of material, in which each phase receives inputs and generates byproducts that can be reused or discarded. In addition, the modeling highlights significant environmental concerns, such as emissions of radioactive dust, sulfur dioxide, and carbon dioxide, reinforcing the need for mitigation strategies. In the mining process, land use represented in purple has a significant impact, followed by water consumption represented in orange. In addition, the high demand for primary energy in gray and transportation in blue highlights the intensity of energy consumption in this phase (see Figure 4).

**Figure 3:**
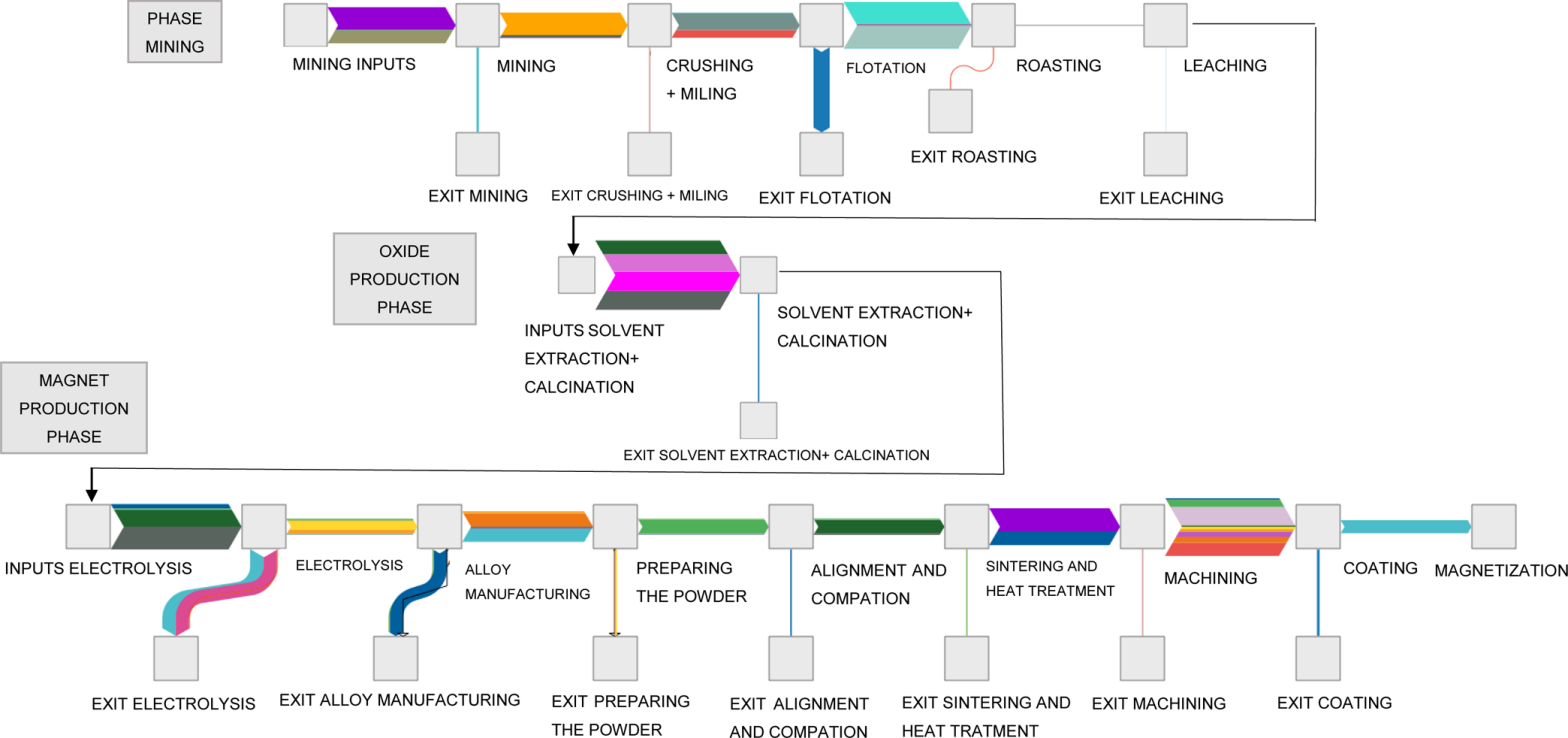
Modeling the production system of 1 kg of Nd–Fe–B magnets.

**Figure 4:**
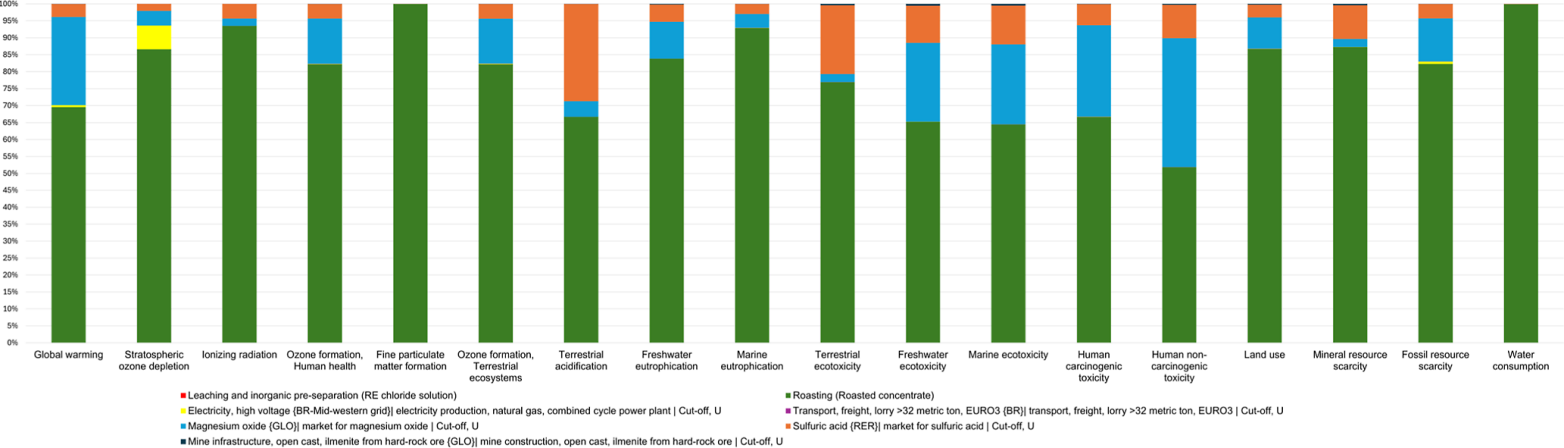
Characterization of the mining phase for the production of 1 kg of Nd–Fe–B magnets using the ReCiPe method.

In this study, the system boundaries and any material production process that contributed 1% to the total weight of the product was excluded, and the mass allocation method was considered. According to the cutting criteria and different manufacturing processes, our analysis includes “cradle to gate” system boundaries, which include all processes from raw material extraction to the production phase.^59,60^

The extraction and calcination of REO involve various inputs, such as electricity, hydrochloric acid (HCl), ammonium hydroxide, and rare-earth fractions classified as medium, heavy, lanthanum, and cerium. The separation of these elements occurs through chemical and physical characteristics, allowing important products to be obtained for use in green technologies. Modeling of the system highlights the importance of energy and chemical reagents as essential inputs in this process. The environmental impact is mainly influenced by the use of HCl in pink, followed by ammonium hydroxide in light purple and electricity in gray, which are critical inputs for the production of rare-earth oxides.

In the modeling of the REM (rare-earth magnets) production system, the phases from material preparation to final magnetization are detailed. The process begins with electrolysis, where oxides of didymium, fluorides, and lime are used to produce Nd–Pr–Fe–B alloys, generating outputs such as gases and liquid waste. Next, the manufacture of the alloy involves melting and mixing the metallic materials, followed by the preparation of the powder by grinding and refining, ensuring proper distribution of the particles. The powder was compacted and aligned to ensure that the particles were oriented correctly, optimizing the magnetic properties of the final magnet. This is followed by sintering and heat treatment, consolidating the powder into a solid block that is then shaped in the machining phase. The magnet is coated with nickel and copper to protect it against corrosion before undergoing the final magnetization process, activating its magnetic properties.

### 2.2 Life-Cycle Inventory Analysis

The LCI consists of identifying and quantifying the resources used in the production of a product, including energy, water, raw materials, and processed materials, as well as the emissions and waste released into the environment, including atmospheric pollutants, discharges into soil and water, and the losses incurred during the production process.^61^ Data for this study were collected from primary and secondary sources. The primary sources included direct measurements and information obtained from the stakeholders of the system analyzed, while the secondary sources consisted of existing databases and the scientific literature.

After validation, the data was entered into the SimaPro 9.1 software, where mass and energy balancing was carried out. Inventory modeling enabled the integration of input and output flows for each unit process, providing a detailed view of the system’s environmental profile. In addition, a critical analysis of the LCI was conducted to identify possible sources of uncertainty and data limitations.

In the context of this research, the LCI for Nd–Fe–B magnets took into account factors such as the energy matrix and transportation logistics. This approach allows for the proposal of strategies to mitigate environmental impacts throughout the entire production chain. In order to validate the system’s data, meetings and technical visits were made to the factory laboratory, allowing for a detailed description of the processes and verification of the mass balances.

### 2.3 Life-Cycle Impact Assessment

Life-cycle impact assessment (LCIA) was performed using SimaPro software in conjunction with the Ecoinvent database and the ReCiPe method, developed by RIVM, Radboud University, CML, and PRé Consultants.^62^

The methodology adopted sought to harmonize the environmental impacts in the models, considering both the midpoint categories, which analyze factors such as global warming, acidification, and eutrophication, and the end point categories, which assess the final impacts on human health, ecosystems, and natural resources. The ReCiPe method quantifies environmental impacts throughout the life cycle of a product or process, covering 17 categories, including global warming, stratospheric ozone depletion, ionizing radiation, ozone formation, form of particulate matter, ozone formation, terrestrial ecotoxicity, freshwater eutrophication, marine eutrophication, terrestrial ecotoxicity, marine ecotoxicity, human carcinogenic, human noncancerous, land use, scarcity of mineral resources, scarcity of fossil resources, water consumption, allowing a comprehensive assessment of environmental effects, human health, and resource consumption.

In addition to ReCiPe, a second impact assessment was carried out using the method of the Intergovernmental Panel on Climate Change (IPCC), which complements the analysis by considering specific indicators, such as the GWP for a 100 year horizon, the consumption of nonrenewable fossil energy, and the full set of ReCiPe end point indicators (Hierarchist). This approach provides a more detailed view of climate change, identifying its causes, effects, and risks for humanity and the environment, contributing to a broader understanding of the environmental impacts of the system assessed.

## 3 Presentation of Results and Discussion

This study addresses the characterization and normalization of environmental impacts using the ReCiPe and IPCC methods, highlighting the main environmental indicators identified in the results.

### 3.1 Characterization in the Mining Phase

The LCA provides a detailed understanding of the environmental impacts associated with mining up to the production of rare-earth magnets, identifying the most critical phases of the production system. As shown in Figure 4, the LCIA characterization results for the mining phase of 1 kg of Nd–Fe–B magnets were obtained using the ReCiPe method.

As illustrated in the figure, the roasting phase, represented in green, stood out as the most impactful in several environmental categories, contributing 99.94% of the impact in the fine particulate matter category and 99.86% in water consumption. In addition, magnesium oxide, represented in navy blue, had significant impacts on human toxicity, accounting for 37.99% of noncarcinogenic toxicity and 26.95% of carcinogenic toxicity, showing environmental and health risks associated with this compound. In the particulate matter category, the mining phase exhibited an emission equivalent to 0.368 kg PM 2.5 eq, indicating a significant potential for fine particle emissions into the atmosphere. These emissions can directly impact air quality and pose health risks, particularly in regions near extraction sites. Water consumption was quantified at 1.53 m^3^, highlighting the mining process’s dependence on water resources. The high demand for water can lead to reduced availability for other uses.

They contribute to alterations in the local aquatic ecosystem. Regarding global warming potential, the mining phase contributed 0.172 kg of CO_2_ eq, reflecting greenhouse gas emissions associated with extraction activities and energy consumption. Although relatively low compared to other life-cycle phases, this impact underscores the need to assess alternatives for reducing carbon emissions in the mining sector.

The results obtained in this study align with the findings of ref 63, which indicate that rare-earth mining can generate significant environmental impacts due to high energy consumption, particulate matter emissions, and the need for large volumes of chemical reagents. According to the authors, rare-earth extraction primarily occurs in carbonatite deposits, ion-adsorption clays, and alkaline rocks, involving energy-intensive processes and generating radioactive waste. In Brazil, REE mining predominantly takes place through open-pit operations, which, while reducing operational costs compared with underground mining, present environmental challenges related to deforestation and intensive water and electricity consumption. Furthermore, the literature highlights that the carbon footprint of rare-earth mining can vary significantly, with projects in shale deposit formations reaching a GWP of up to 229 t CO_2_-eq/t-TREO.

Another relevant aspect of the analysis is the comparison with the studies by,^64^ which evaluated the extraction of REO from ion-adsorption clays. The authors highlight that the production of 1 kg of HREOs can result in emissions ranging from 258 to 408 kg CO_2_-eq, in addition to requiring between 270 and 443 MJ of primary energy. The mining and extraction phase is the primary contributor to environmental impacts due to the intensive use of ammonium sulfate for leaching and the high electricity demand. Ammonium sulfate stands out as one of the major contributors to impact categories such as terrestrial acidification and eutrophication, reinforcing the need for strategies to minimize its use in the process. Furthermore, the reliance on China’s coal-based energy matrix increases emissions associated with electricity consumption, highlighting the importance of developing more efficient and sustainable methods for rare-earth mining.

### 3.2 Characterization of the Rare-Earth Oxide Production Phase

For the characterization of the oxide production phase, Figure 5 was developed to illustrate the impact categories and identify the phases that contribute the most to the environmental impacts.

**Figure 5:**
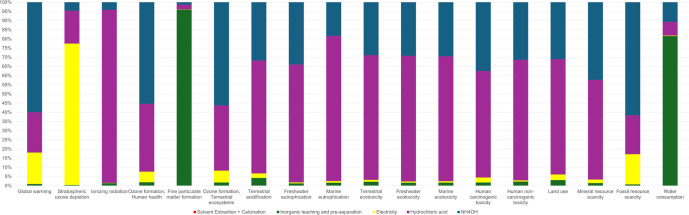
Characterization of the rare-earth oxide production phase for the production of 1 kg of Nd–Fe–B magnets using the ReCiPe method.

The production of REOs involves energy- and chemical-intensive processes, resulting in significant environmental impacts. The leaching and preseparation phase, highlighted in the analysis with light green, showed the highest impacts in the fine particulate matter formation category (95.96%) and water consumption (81.57%), represented in dark blue, indicating a high demand for water resources. Additionally, the chemical compound hydrochloric acid exhibited a substantial influence across various environmental categories, represented in yellow, contributing 94.39% to the ionizing radiation category and 79.21% to marine eutrophication. These results align with the findings of ref 63, which emphasize that oxide refining is the most impactful phase of the production chain, primarily due to the intensive use of electricity and chemical reagents. Similarly, ref 65 analyzed CO_2_-eq emissions throughout the REE.

The study revealed that, in the solvent extraction and electrolytic refining phases, chemical reagents such as neodymium fluoride, lithium fluoride, and electricity become the main contributors to environmental impact. These observations highlight the need to optimize input usage and adopt lower impact technologies in REO production. The solvent extraction method, widely used for rare-earth-element separation, presents challenges due to its high demand for solvents and strong acids, which can contaminate water resources and generate large volumes of chemical waste.

### 3.3 Characterization of the Rare-Earth Magnet Production Phase

For the Nd–Fe–B magnet manufacturing phase, graphical representations were created to show the categories and environmental impacts associated with the process. Figure 6 illustrates the characterization of environmental impacts throughout the different production phases, from the mining of rare-earth magnets to the magnet production phase.

**Figure 6:**
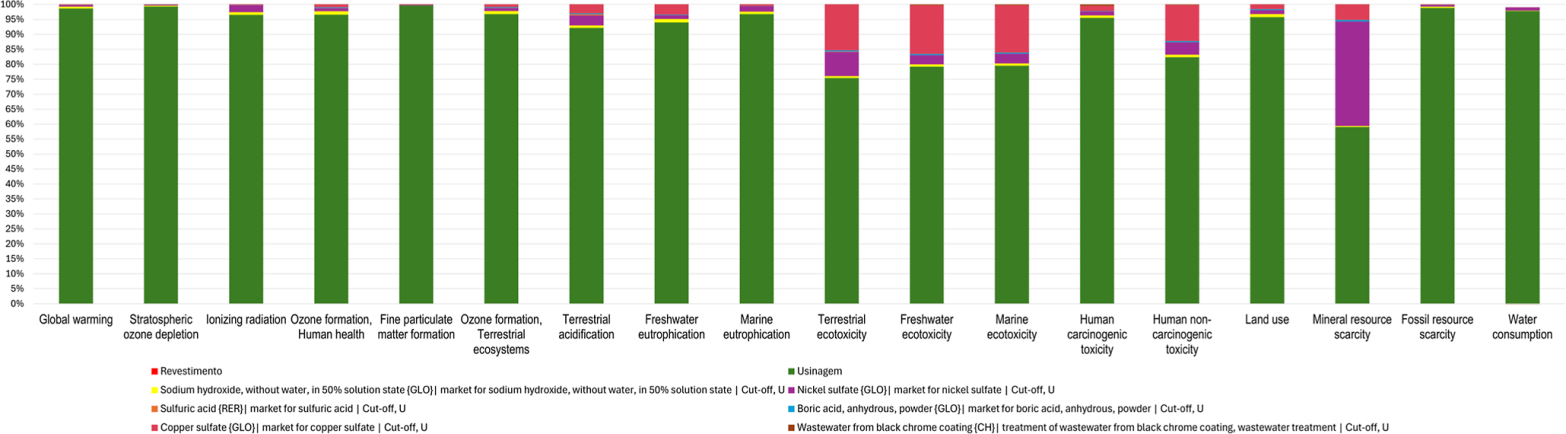
Characterization of the Nd–Fe–B magnet production phase for 1 kg using the ReCiPe method.

As observed, machining, highlighted in green, had a significant influence on all of the categories analyzed, accounting for 98.68% of the emissions associated with global warming, 99.32% of the stratospheric ozone depletion, and 98.69% of water consumption. Additionally, nickel sulfate, represented in yellow, had a significant impact on the mineral resource scarcity category, contributing to 34.92%. The results of this research are in line with the findings of ref 65, who analyzed the CO_2_-eq emission network throughout the manufacturing and recycling of Nd–Fe–B permanent magnets. The study identified that Nd–Fe–B magnets, steel, and ferroboron showed the highest dependence on CO_2_-eq emission flows, while recycled REE and glucose had a lesser influence on the propagation of these emissions. Additionally, the Nd–Pr alloy, electricity, and steel were identified as critical in magnet manufacturing, while glucose, diesel, and electricity were more central to the recycling of these materials. Regarding the global warming potential, magnetite

manufacturing resulted in 212 kg of CO_2_ eq. This result highlights the significance of greenhouse gas emissions associated with rare-earth processing, reinforcing the need to evaluate alternatives for carbon emission reduction in the sector. In the aquatic ecotoxicity category, copper sulfate contributed 16.3% to freshwater ecotoxicity and 15.8% to marine ecotoxicity. Nickel sulfate, in turn, contributed 34.9% to the mineral resource scarcity category, highlighting its influence on the availability of the essential metals. Furthermore, rare-earth magnet manufacturing resulted in an emission of 11.3 kg of 1,4-DCB (direct characterization at biosphere) in the marine ecotoxicity category, illustrating the environmental impact associated with the release of toxic substances during the industrial process.

The literature emphasizes that Nd–Fe–B magnet production is a process that is highly intensive in energy and chemical inputs. According to ref 63, the energy consumption in REE metallurgy surpasses that of conventional metals such as copper and aluminum due to the complexity of the purification processes. In addition to atmospheric emissions associated with fossil fuel combustion, there are additional environmental risks related to chemical contamination from the solvents used in metal separation.

### 3.4 Normalization of the Production of 1 kg of Nd–Fe–B Magnets

The normalized results, as shown in Figure 7, indicated that the machining phase contributed to the human toxicity, freshwater ecotoxicity, and marine ecotoxicity impact categories. Copper sulfate was identified as one of the main contributors in these categories, being the second most relevant element in terms of environmental impact. These data highlight the need for control and optimization measures in machining to minimize its influence on the life cycle of the REM. The results indicate the significance of the mining, oxide production, and rare-earth magnet manufacturing phases in generating environmental impacts and emphasize the importance of mitigation strategies, such as the adoption of cleaner technologies, water reuse, and process optimization. Future studies could explore measures to reduce the environmental footprint through alternative, sustainable production scenarios.

**Figure 7:**
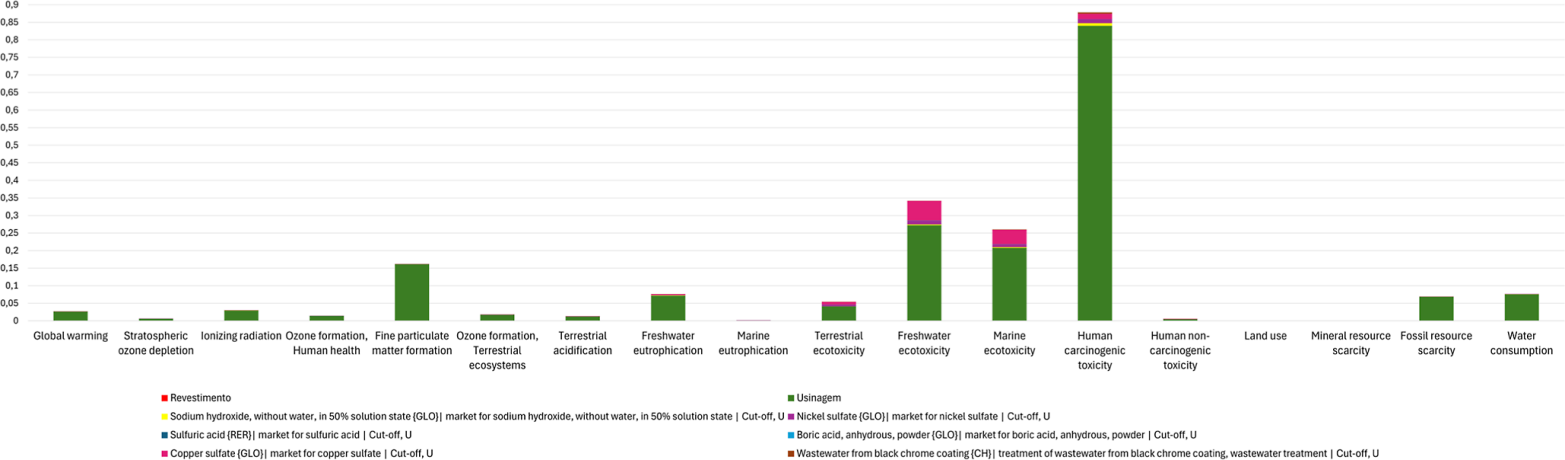
Normalization of rare-earth magnet production using the ReCiPe method.

### 3.5 Nd–Fe–B Magnet Production Using the IPCC Method

The results obtained by using the IPCC method are presented in Table 1. The production of rare-earth magnets resulted in impacts of 207 kg of CO_2_ eq in the fossil GWP category, 0.211 kg of CO_2_ eq in the biogenic GWP category, and 0.133 kg of CO_2_ eq in the land transformation category. These results underscore the need for strategies to mitigate the environmental impacts of magnet manufacturing with a particular focus on reducing greenhouse gas emissions and minimizing land use impacts.

**Table 1:** Results for the Production of 1 kg of Nd–Fe–B Magnets, Assessed Using the IPCC Method

	Fossil	Biogenic	land transformation
GWP	207 kg of CO_2_ eq	0.211 kg of CO_2_ eq	0.134 kg of CO_2_ eq

To ensure the robustness of the results, the ReCiPe and IPCC methods were used in the uncertainty analysis. As highlighted by,^66^ uncertainty analysis in LCA is essential for assessing the robustness of the results, since it accounts for the variabilities and limitations inherent in the inventory data and the models used. The results obtained indicated that, in the climate change category, the ReCiPe method yielded a value of 1.08 × 10^8^ kg CO_2_ eq, while the IPCC method resulted in 1.06 × 10^8^ kg CO_2_ eq, corresponding to a relative variation of 1.86%, which demonstrates consistency between the approaches. Thus, the importance of applying multiple LCA methods is highlighted, since the use of different methodological approaches contributes to the cross-validation of results and to increasing the reliability of the conclusions obtained.

### 3.6 Toxicity of Elements in Rare-Earth Magnet Production and Their Impacts on Human Health

Rare-earth magnet production involves several phases, from mining to final manufacturing, requiring the extraction and processing of a wide range of minerals and heavy metals. Many of these elements have adverse effects on human health depending on the form of exposure, concentration, and duration of contact. Workers involved in this production cycle are particularly susceptible to occupational hazards, while the release of these metals into the environment can pose a long-term public-health problem.

This section presents Table 2, outlining the presence of inputs throughout the rare-earth magnet production cycle, from ore extraction to final manufacturing. Additionally, inventory data were compared with three recent studies analyzing the toxicity of these elements and their impacts on the human body. This approach allows a detailed assessment of the risks associated with each phase of the production process, which reinforces the need for environmental and occupational mitigation strategies.

**Table 2:** Chemical Elements

element	flow type	compartment	value	unit
arsenic	emission	air	96.4	mg
arsenic	emission	water	439	mg
arsenic	emission	soil	787	μg
tin	input	raw material	354	mg
tin	emission	air	81	μg
tin	emission	water	1.56	mg
tin	emission	soil	4.38	μg
scandium	input	raw material	263	μg
scandium	emission	air	916	μg
scandium	emission	water	630	μg
scandium	emission	soil	135	μg
Lanthanium-140	input	raw material	596	g
Lanthanium-140	emission	air	464	μBq
Lanthanium-140	emission	water	17.3	mBq
Cerium-141	emission	water	6.44	MBq
Cerium-141	emission	soil	1.32	mBq
copper	input	raw material	220	g
nickel	input	raw material	99.8	g
dysprosium	input	raw material	25.7	g
neodymium	input	raw material	437	g
praseodymium	input	raw material	116	g
samarium	input	raw material	77.2	g

Recent studies highlight the relevance of metal and REE toxicity to human health. The study^67^ investigated the presence of metals and REE in healthy and tumor tissues from the mammary glands of dogs, used as a model for human impacts. The results indicated that metals such as copper (Cu) and molybdenum (Mo) showed high concentrations in tumor tissues, which were associated with oxidative stress and inflammatory processes related to cancer development. Furthermore, lead (Pb), thallium (Tl), arsenic (As), and mercury (Hg) were identified as neurotoxic and carcinogenic in addition to being linked to kidney and cardiovascular diseases. REE, on the other hand, was found more frequently in healthy tissues than in tumor tissues, suggesting a possible protective relationship or influence on tumor progression.

In the study,^68^ the environmental and human health impacts of mining and processing critical minerals in Indonesia were analyzed, including nickel (Ni), gold (Au), copper (Cu), tin (Sn), and bauxite (Al_2_O_3_). Ni was associated with respiratory diseases such as asthma and bronchitis as well as potential carcinogenic effects. Au, Sn, and Cu were identified as metals with hepatic and neurological toxicity, whereas aluminum (Al) was linked to respiratory and neurological diseases. Furthermore, elements such as Pb, Hg, As, and chromium (Cr) were associated with elevated risks of cancer, kidney damage, and neurotoxicity.

On the other hand, the study^69^ reviewed toxicological studies on the impacts of rare-earth elements on occupational health, covering workers exposed to mining, transportation, and manufacturing of materials containing these elements. The results indicate that scandium (Sc), yttrium (Y), lanthanum (La), cerium (Ce), and neodymium (Nd) tend to cause pulmonary and hepatic toxicity. In contrast, praseodymium (Pr), samarium (Sm), europium (Eu), and gadolinium (Gd) may have toxic effects on kidneys. Elements such as terbium (Tb), dysprosium (Dy), holmium (Ho), erbium (Er), thulium (Tm), ytterbium (Yb), and lutetium (Lu) show potential for metabolic and respiratory impacts, although they have been poorly studied.

The data analysis highlights the significant presence of heavy metals and rare-earth elements in various industrial and environmental contexts, with adverse impacts on human health, including lung diseases, cancer, and neurological and kidney disorders. Occupational exposure to these elements requires stringent safety measures to reduce health risks for workers, while the release of these compounds into the environment remains a critical challenge for public health and environmental sustainability.

The recycling of Nd–Fe–B permanent magnets results in significant environmental benefits compared with the primary production route involving rare-earth mining. According to the literature on LCA, magnet-to-magnet recycling processes can reduce global warming potential by approximately 18% to 33% in conservative scenarios, primarily due to the elimination of the mineral extraction and chemical separation stages of rare-earth elements.^70^ In more comprehensive comparative analyses, it is observed that the overall reduction in environmental impacts can reach between 64% and 96% across multiple impact categories, including primary energy consumption, human toxicity, ecotoxicity, and depletion of abiotic resources, depending on the recycling method and the modeling assumptions adopted.^71^ These gains are explained by the fact that the primary production of Nd–Fe–B magnets is heavily dependent on energy- and chemical-reagent-intensive processes, especially in the mining and separation stages of neodymium and other critical elements, which are avoided or significantly reduced in recycling.^72^ Thus, the recycling of rare-earth magnets emerges as a robust environmental mitigation strategy with the potential to significantly reduce impacts in virtually all LCA categories evaluated.

### 3.7 Limitations of the Present Study

This study has some limitations inherent to the methodology used and the availability of data that may influence the accuracy of the results. One of the main restrictions is related to the scarcity of detailed primary data on rare-earth magnet production in Brazil. As a result, it was necessary to rely on secondary data sources and information from international studies, which may limit the representativeness of the environmental impacts specific to the national context.

Furthermore, the adaptation of LCA inventories to reflect the Brazilian reality required the use of estimates and approximations, particularly with regard to energy consumption and chemical inputs. The lack of a robust national LCA database limits the accuracy of process modeling, as impact coefficients may not fully reflect the local conditions.

Given the lack of a consolidated national database for LCA in the mining sector, secondary data from Ecoinvent was used to model the underlying processes, including the supply of energy and other inputs, with appropriate adjustments to reflect the Brazilian energy mix. Data quality was assessed using the pedigree matrix, considering the criteria of accuracy, completeness, and representativeness.^73^ The system modeling was subsequently refined based on inventory data, integrating information from Ecoinvent and supplementing it with data from the specialized literature to ensure the inventory’s adherence to Brazilian conditions. It should be noted that some of the information used is classified and confidential.

Another limitation concerns the scope of the study, which considered phases from mining to magnet manufacturing but did not include aspects such as the distribution, use, and final disposal. While the focus was on production, the life-cycle analysis could be expanded to assess impacts over a broader horizon, considering the circular economy and the recycling potential of Nd–Fe–B magnets.

Finally, the uncertainties associated with the environmental impact assessment methodologies should also be highlighted. The choice of the ReCiPe method and the EcoInvent database provides a detailed analysis but may not fully capture all regional variables and specific dynamics of the rare-earth magnet production sector in Brazil. Therefore, future research could aim to enhance national databases, integrate new impact assessment methodologies, and include additional phases of the production chain for a more comprehensive view of the sustainability of these materials.

## 4 Final Considerations

Brazil is currently undergoing a significant regulatory transition regarding the governance of critical minerals, particularly rare-earth elements used in the Nd–Fe–B magnet supply chain. The National Congress is currently evaluating the proposal to establish the National Policy on Critical and Strategic Minerals (PNMCE) through Bill 2780/2024, which introduces planning instruments, incentives for industrialization, and guidelines aimed at promoting the vertical integration of the mineral value chain. This policy explicitly recognizes rare-earth elements as strategic resources for the energy transition and national technological development. In parallel, complementary proposals reinforce the need for value addition within the country, including requirements for domestic processing and industrialization of strategic minerals, traceability mechanisms, and potential restrictions on the export of unprocessed materials.

This regulatory movement reflects an institutional acknowledgment that Brazil still operates predominantly as an exporter of raw materials, with limited integration of critical stages such as separation, refining, and metallization, which are key processes in the rare-earth magnet supply chain. In this context, the absence of a fully structured industrial chain suggests that LCA studies based solely on generic international databases may not accurately represent the Brazilian reality, particularly with regard to mining, chemical processing, and logistics inventories. Therefore, the incorporation of primary national data or the adaptation of international data sets to local conditions becomes essential to ensure geographical representativeness, reduce uncertainties, and enhance the methodological robustness of LCA studies applied to rare-earth systems in Brazil.

Within this context, this study analyzed the life cycle of Nd–Fe–B magnet production, encompassing the stages of mining, oxide production, and magnet manufacturing with a focus on the Brazilian scenario. The results indicate that all phases of the production chain are associated with significant environmental impacts, highlighting the need for mitigation strategies to support more-sustainable production pathways.

The mining phase was identified as one of the most impactful stages, mainly due to high energy and water consumption as well as emissions of fine particulate matter and the generation of toxic waste. Additionally, the roasting process, which is essential for the extraction of rare-earth elements, contributed significantly to overall environmental impacts, particularly in categories related to particulate emissions and water resource use.

In the oxide production stage, leaching and preseparation emerged as the most critical processes, driven by high water consumption and the intensive use of chemical reagents such as hydrochloric acid. These processes resulted in significant contributions to impact categories, such as marine eutrophication and ionizing radiation, emphasizing the need for strategies aimed at reducing the use of aggressive chemicals and minimizing waste generation.

Finally, the magnet manufacturing stage also presented considerable environmental impacts, with machining identified as a major contributor to global warming, ozone-layer depletion, and water consumption. Furthermore, machining and coating processes highlight the importance of adopting more sustainable practices, including material efficiency, reuse strategies, and integration of recycling technologies.
